# The turnover of continental planktonic diatoms near the middle/late Miocene boundary and their Cenozoic evolution

**DOI:** 10.1371/journal.pone.0198003

**Published:** 2018-06-05

**Authors:** Tatsuya Hayashi, William N. Krebs, Megumi Saito-Kato, Yoshihiro Tanimura

**Affiliations:** 1 Department of Environmental Changes, Faculty of Social and Cultural Studies, Kyushu University, Motooka, Nishi-ku, Fukuoka, Japan; 2 Mifune Dinosaur Museum, Mifune, Kumamoto, Japan; 3 Independent Researcher, Round Top, Texas, United States of America; 4 Department of Geology and Paleontology, National Museum of Nature and Science, Amakubo, Tsukuba, Ibaraki, Japan; University of Nottingham, UNITED KINGDOM

## Abstract

Fossil evidence indicates that modern assemblages of temperate nonmarine planktonic diatoms began near the middle/late Miocene boundary when the genus *Actinocyclus*, an important constituent of lacustrine planktonic diatom assemblages during the early to middle Miocene, was replaced by genera of the family Stephanodiscaceae. This floral turnover has been confirmed in many regions of the world, except eastern Asia where taxonomic data about early and middle Miocene planktonic diatom assemblages have until recently been scarce. Our analysis of Lower and Middle Miocene lacustrine diatomaceous rocks in Japan confirms that species of nonmarine *Actinocyclus* were important constituents of lake phytoplankton there as well. The appearance of nonmarine *Actinocyclus* species near the beginning of the Miocene may have resulted from the introduction of euryhaline species into lacustrine environments during a highstand of sea level at that time. Similarly, it is possible that species of Stephanodiscaceae evolved from marine thalassiosiroid ancestors that invaded high latitude lacustrine environments during multiple Paleogene highstands, resulting in a polyphyletic origin of the family. The turnover from nonmarine *Actinocyclus* to Stephanodiscaceae genera near the middle/late Miocene boundary may be linked to a contemporaneous increase in silica concentrations in lakes caused by active volcanism, increased weathering of silicate rocks due to orogeny, and the expansion of C_4_ grasslands. This turnover may also have been influenced by enhanced seasonal environmental changes in the euphotic zone caused by the initiation of monsoon conditions and a worldwide increase in meridional temperature gradients during the late Miocene. Morphological characteristics of Stephanodiscaceae genera, such as strutted processes and small size, suggest their species were better adapted to seasonal environmental changes than nonmarine species of *Actinocyclus* because of their superiority in floating and drifting capabilities and possibly metabolism, intrinsic growth rate, and reproductivity. As climates deteriorated during the late Miocene, Stephanodiscaceae species may have spread from high latitudes to temperate lakes where they diversified, ultimately displacing *Actinocyclus*.

## Introduction

The Miocene was a significant time in the evolution of continental planktonic diatoms [1–6] that witnessed two salient events: the colonization and diversification of the marine genus *Actinocyclus* Ehrenberg into temperate lacustrine systems during the early to middle Miocene, and the turnover from a flora characterized by nonmarine *Actinocyclus* to one dominated by genera of the family Stephanodiscaceae Glezer & Makarova (*Cyclotella* (Kützing) Brébisson, *Cyclostephanos* Round, *Discostella* Houk & Klee, *Lindavia* (Schütt) De Toni & Forti, *Mesodictyon* Theriot & Bradbury, *Stephanodiscus* Ehrenberg, *Tertiarius* Håkansson & Khursevich, etc.) near the middle/late Miocene boundary [1–5]. After the latter event, genera of Stephanodiscaceae diversified and are now important and characteristic constituents of phytoplankton in temperate lake environments.

Biochoronological data collected from North and South America, Europe, Africa, and northern Asia over the last forty years [1, 2, 7–23] have consistently supported this succession of lacustrine diatom floras from *Actinocyclus* in the early and middle Miocene to genera of the Stephanodiscaceae in the late Miocene (see the discussion section). For a very large part of Asia (except northern Asia), however, taxonomic data on Miocene nonmarine planktonic diatom floras have been very limited. Recently, the allochthonous occurrences of Stephanodiscacae genera have been reported from Upper Miocene marine outcrops in Japan [24], and the dominance of *Actinocyclus* has been confirmed in Lower Miocene lacustrine samples dredged from the Yamato Rise and the Ulleung Plateau in the Japan Sea [25, 26].

Aside from the evolution of nonmarine planktonic diatoms, the Miocene was also a time when regime shifts of climatic and environmental conditions occurred on a global scale, with many of them (e.g., development of monsoons and expansion of C_4_ grasses) originating in Asia (e.g., [27]). Therefore, data on fossil nonmarine planktonic diatoms in Asia during the Miocene are necessary to understand the linkage between the late Cenozoic evolution of continental planktonic diatoms and climatic and environmental changes.

In this study, we report on the discovery of additional species of nonmarine *Actinocyclus* species in Lower to Middle Miocene lacustrine deposits in Japan (eastern Asia). Although some of the fossil nonmarine *Actinocyclus* species may be new, their description is beyond the scope of this study and will be done in the future. The purpose of this work is to present possible causal linkages between the evolution of nonmarine planktonic diatoms and the contemporaneous climatic and environmental changes that occurred during the Miocene.

## Materials and methods

**Fig 1:**
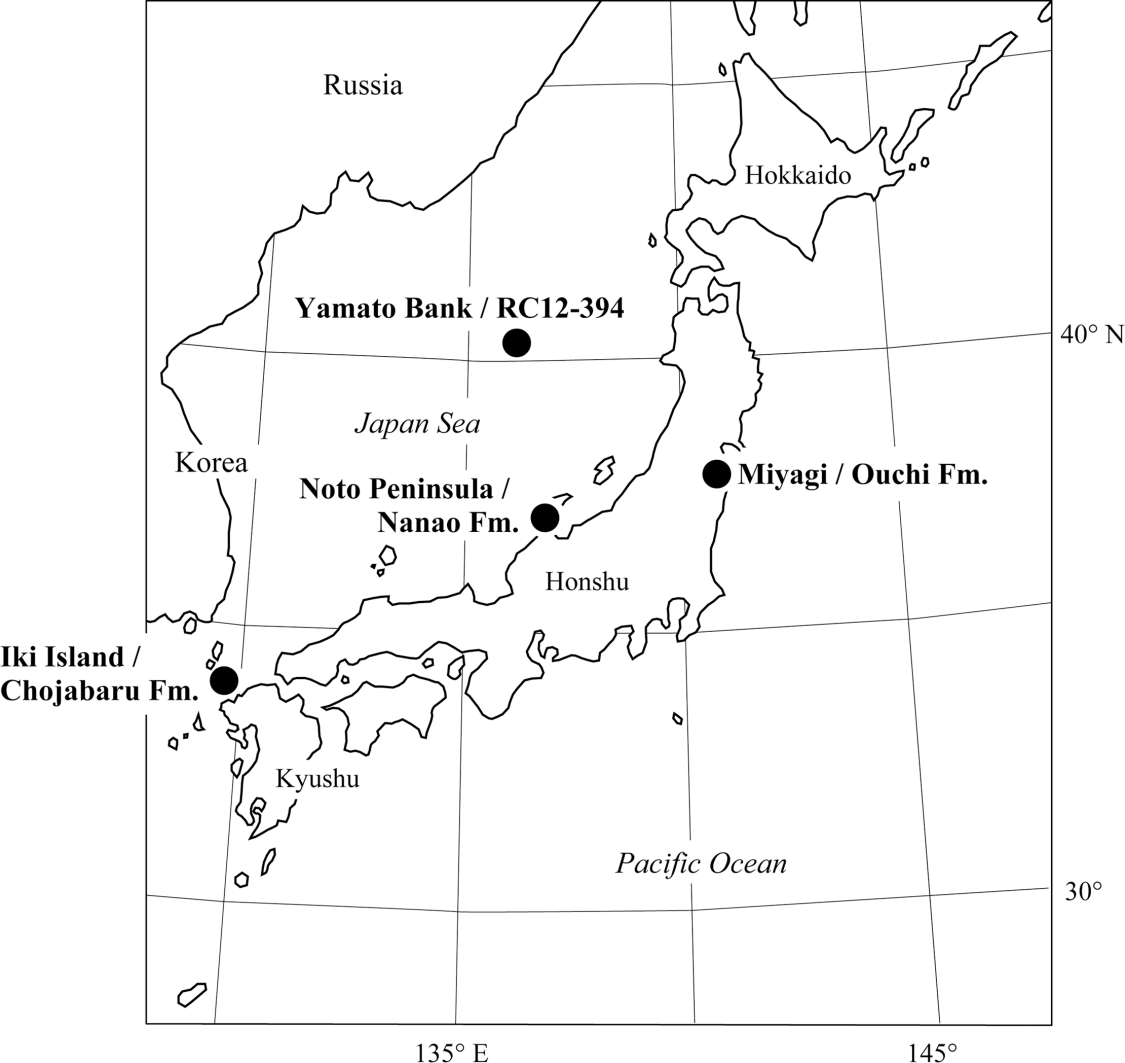
Localities with nonmarine Miocene species of *Actinocyclus* in Japan.

**Fig 2:**
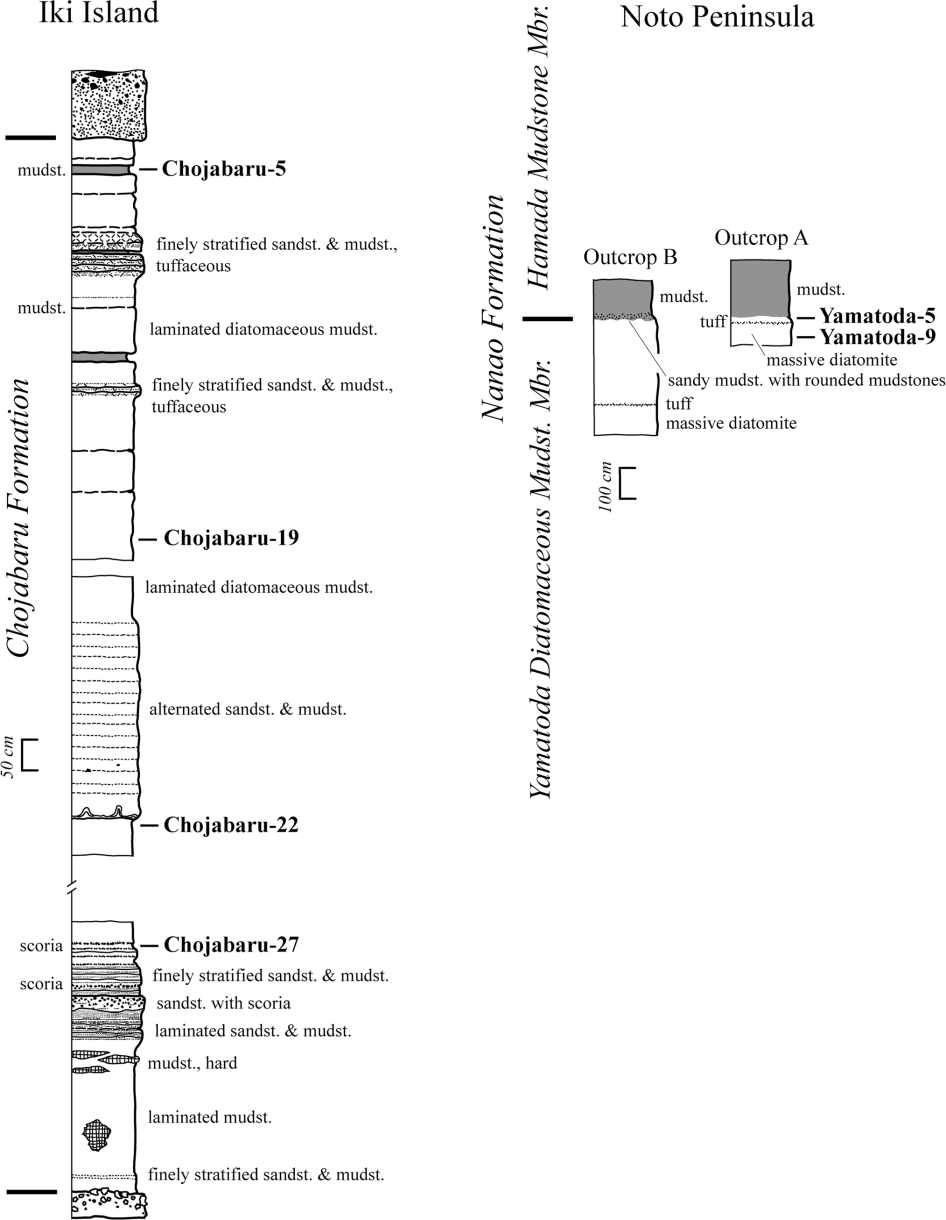
Stratigraphic columnar sections at Iki Island and Noto Peninsula showing stratigraphic position of samples studied. The position of these sections does not indicate their correlation.

We examined seven samples collected from three Miocene freshwater diatomites in Japan (Figs 1 and 2): two samples from the Yamatoda Diatomaceous Mudstone Member of the Nanao Formation (Yamatoda, Nakajima-machi, Ishikawa Prefecture: 37°7.45'N, 136°49.8'E) [28], four samples from the Chojabaru Formation (Yawata-ura, Ashibe-cho, Nagasaki Prefecture: 33°46.8'N, 129°49.8'E) [29], and one sample from the Ouchi Formation (Ouchi, Marumori-machi, Miyagi Prefecture: 37°52.5'N, 140°49.5'E) [30]. The board of education of Ashibe Town (present Iki City), Nagasaki Prefecture, and Taniguchi Braiding Co., Ltd., Ishikawa Prefecture, gave us permits to obtain mudstone samples from several outcrops of Chojabaru and Nanao Formations, respectively. The outcrops did not involve endangered or protected species. All the formations yield Daijima-type floras, a mixture of deciduous and evergreen broad-leaved trees with conifers [31], which are widely found on Japanese islands and the Japan Sea side of Korea, Sakhalin and Siberia [32]. This flora was reported from the Yamatoda Diatomaceous Mudstone Member (Nanao Formation) [33], while the fossil flora found in the Chojabaru Formation was assigned to a probable Daijima-type [34], and this flora was reported from the Ouchi Formation [35]. An earliest Miocene to early middle Miocene age (ca. 22–14 Ma or 13 Ma) was assigned to these formations based on radiometric ages and biostratigraphic data [36], and hence the ages of the samples used in this study are likely between 22 and 13 Ma (early to middle Miocene).

A freshwater diatomite in the deep-sea core RC12-394 recovered from the northeast flank of Yamato Bank (40°19’N, 136°13.5’E, water depth 2338 m, Fig 1), the central part of the Japan Sea, was also examined in this study. The core contains 3.5-m-thick diatomite of freshwater origin at the bottom, and a 2.5-m-thick mudstone of marine origin in the upper part. We used three samples taken from the lower diatomite to take light microscope (LM) and scanning electron microscope (SEM) photographs of *Actinocyclus nipponicus* Hayashi, Saito-Kato & Tanimura and *A*. *bradburyii* Hayashi, Saito-Kato & Tanimura, which were recently described [37]. It is suggested the freshwater diatomite was very likely early Miocene in age for two reasons: close similarity in diatom assemblages between the freshwater diatomite in the RC12-394 core to those in several early Miocene freshwater diatomite formations in Japanese islands, and the difference in diatom assemblages between the diatomite and the latest Miocene sediments found in several piston cores taken very close to RC12-394 [38, 39]. No genera of Stephanodiscaceae were found in core RC12-394.

Sample preparation for LM and SEM observations followed the procedure described in [40]. LM and SEM observations of fossil diatoms were performed with the use of a Nikon^®^ ECLIPSE 80i (Kyushu University, Japan) and a JEOL^®^ JSM-5310 (National Museum of Nature and Science, Japan). Morphological terminology follows [41]. In each sample, at least 300 specimens were counted under LM observation and then relative abundances were calculated (Table 1). All samples used in this study are stored in Department of Geology and Paleontology, National Museum of Nature and Science, Tsukuba, Japan.

**Table 1:** Relative abundances of nonmarine diatom species from Lower to Middle Miocene lacustrine deposits in Japan.

	*Actinocyclus* sp. 1	*Actinocyclus* sp. 2	*Actinocyclus* sp. 3	*Actinocyclus* sp. 4	*A*. *nipponicus*	*A*. *bradburyii*	*Aulacoseira* spp.	pennates
Yamatoda-5	44.4%	0.0%	0.0%	0.0%	0.0%	0.0%	55.0%	0.6%
Yamatoda-9	4.5%	0.0%	0.0%	0.0%	0.0%	0.0%	95.2%	0.3%
Chojabaru-5	0.0%	0.0%	4.6%	0.0%	0.0%	0.0%	92.9%	2.5%
Chojabaru-19	0.0%	81.6%	0.0%	0.0%	0.0%	0.0%	17.5%	0.9%
Chojabaru-22	0.0%	99.4%	0.0%	0.0%	0.0%	0.0%	0.3%	0.3%
Chojabaru-27	0.0%	62.5%	0.0%	0.0%	0.0%	0.0%	0.6%	36.9%
Ouchi-1	0.0%	0.0%	0.0%	44.3%	0.0%	0.0%	52.0%	3.7%
RC12-394-240	0.0%	0.0%	0.0%	0.0%	8.3%	0.3%	89.1%	2.4%
RC12-394-260	0.0%	0.0%	0.0%	0.0%	5.2%	1.5%	91.1%	2.1%
RC12-394-490	0.0%	0.0%	0.0%	0.0%	6.6%	1.0%	90.9%	1.6%

In this study, we pay particular attention to the genus *Actinocyclus* and also to genera of the family Stephanodiscaceae. Stephanodiscaceae includes the genera *Cyclotella*, *Cyclostephanos*, *Discostella*, *Lindavia*, *Mesodictyon*, *Stephanodiscus*, *Tertiarius*, and others. Many species of *Lindavia* and *Discostella* were formerly classified within the genus *Cyclotella*. *Cyclotella* species were transferred from the so-called *comta* group [42] to the genus *Puncticulata* Håkansson by [43], but later, some of *Puncticulata* species were transferred into the genus *Handmannia* Peragallo because of the nomenclatural priority of *Handmannia* over *Puncticulata* by [44]. Furthermore, recently some *Handmannia* (*Puncticulata*) taxa with *Pliocaenicus* Round & Håkansson taxa were re-transferred into the genus *Lindavia* for nomenclatural and diagnostic reasons [45]. Because *Lindavia* has priority over *Puncticulata* and *Handmannia* according to [45], many taxa are not yet transferred. As recognized by [45], some genera require additional analysis to examine their relationship to *Lindavia*, and it is possible that *Lindavia* will be divided into multiple genera based on morphological and/or molecular analyses. Since these taxonomic revisions are irrelevant to the purpose of this paper, all genera belonging to the family Stephanodiscaceae, except *Cyclostephanos*, *Stephanodiscus* and *Mesodictyon*, will herein be referred to as cyclotelloids.

## Results

**Fig 3:**
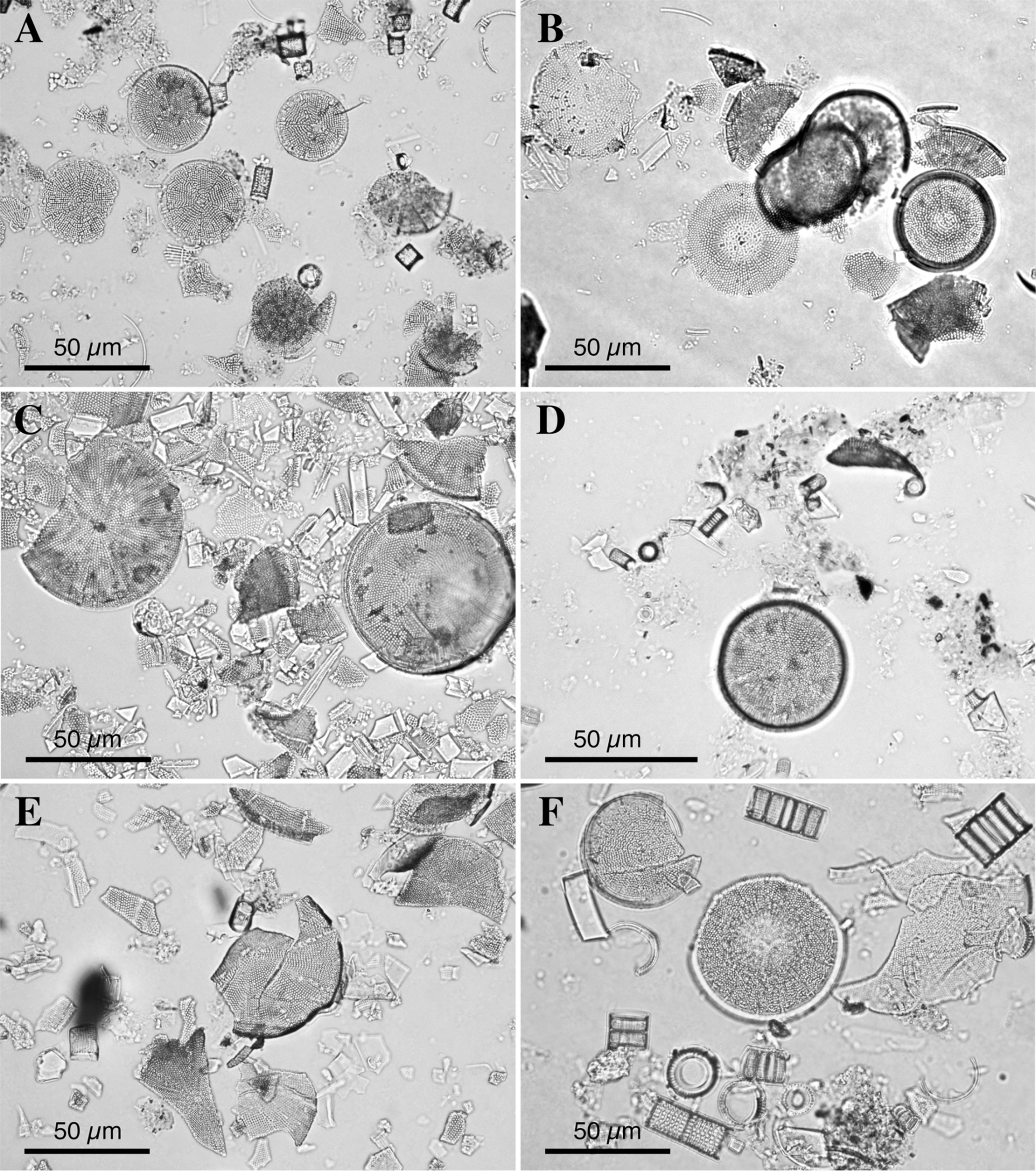
Typical assemblages observed in individual sediment samples. (**A**) *Actinocyclus* sp.1 and *Aulacoseira* spp. from the Yamatoda Diatomaceous Mudstone Member (Yamatoda-5). (**B**) *A*. sp. 2 from the Chojabaru Formation (Chojabaru-22). (**C**) *A*. sp. 2, *Aulacoseira* spp. and penates from the Chojabaru Formation (Chojabaru-27). (**D**) *A*. sp. 3 and *Aulacoseira* spp. from the Chojabaru Formation (Chojabaru-5). (**E**) *A*. sp. 4 and *Aulacoseira* spp. from the Ouchi Formation (Ouchi-1). (**F**) *A*. *bradburyii* (upper left), *A*. *nipponicus* (center) and *Aulacoseira* spp. from the RC12-394 core (RC12-394-490).

**Fig 4:**
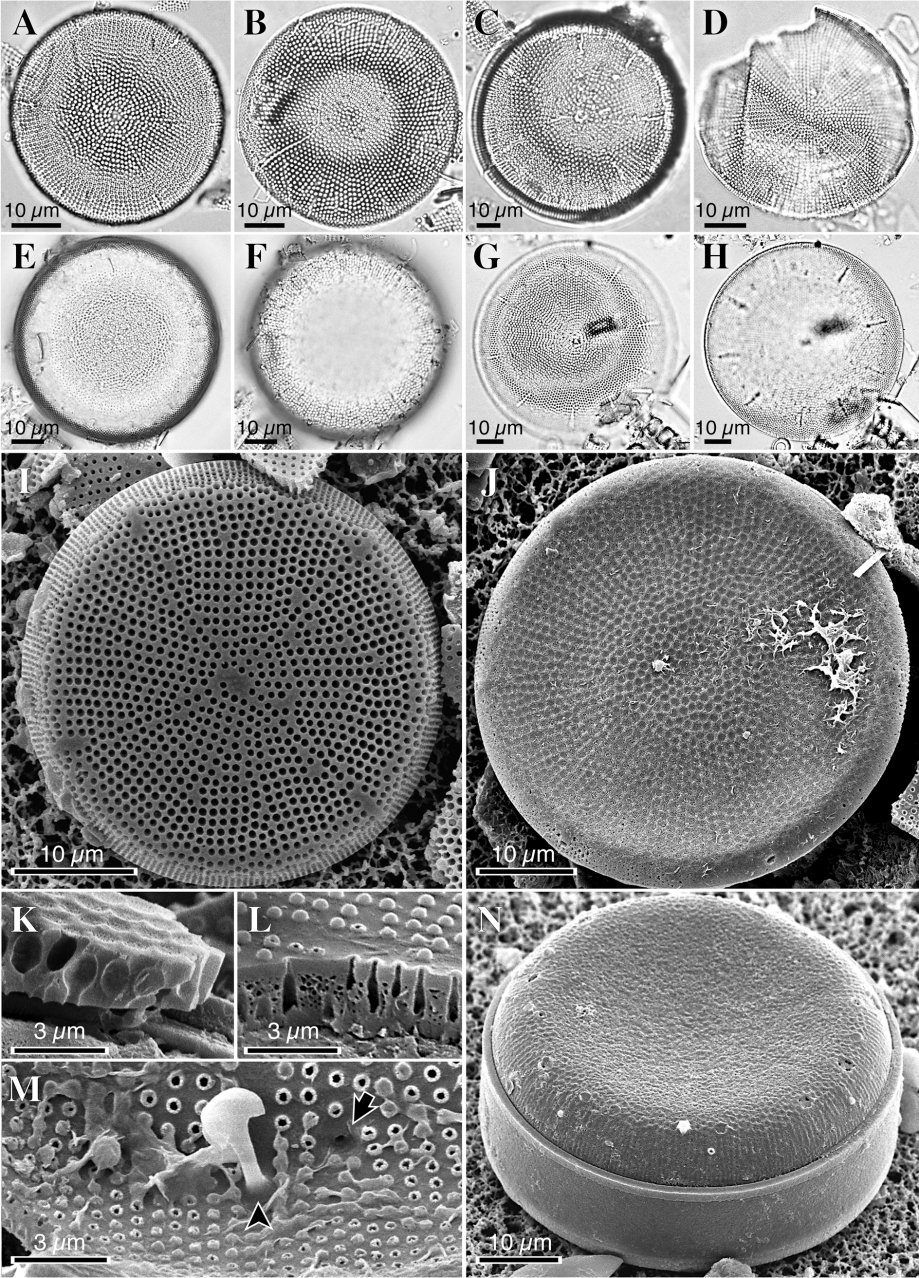
Morphology of *Actinocyclus* species from early to middle Miocene lacustrine sediments in Japan. LM photographs show (**A**) *A*. sp. 1, (**B**) *A*. sp. 2, (**C**) *A*. sp. 3, (**D**) *A*. sp. 4, (**E**, **F**) *A*. *nipponicus* (the same specimen at different focal planes) and (**G**, **H**) *A*. *bradburyii* (the same specimen at different focal planes). SEM photographs show (**I**) *A*. sp. 1, (**J, K**) *A*. sp. 2 and (**L, M, N**) *A*. sp. 3. In **M**, a black arrow and a black arrowhead indicate a pseudonodulus and a labiate process, respectively.

**Fig 5:**
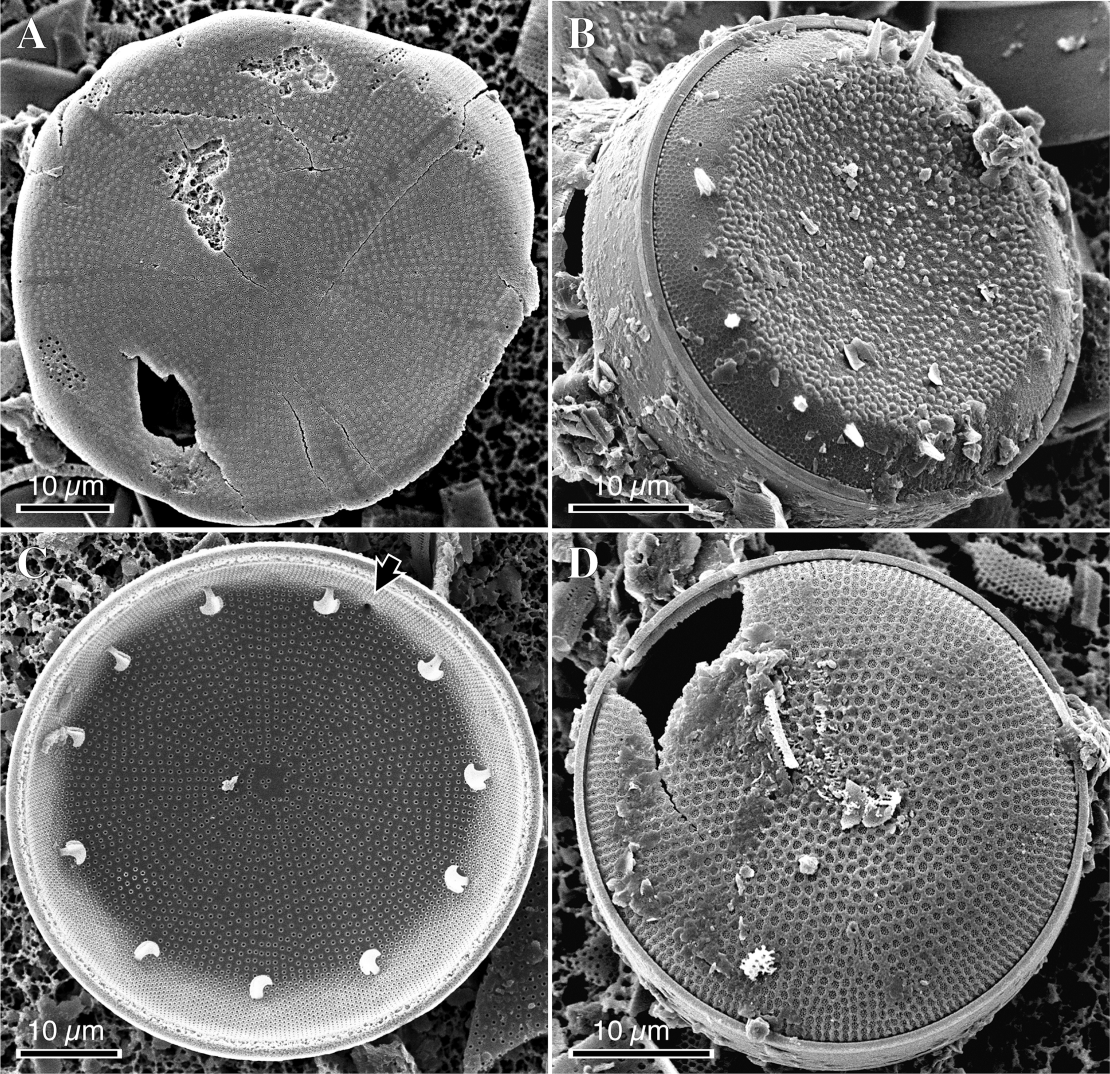
SEM photographs of *Actinocyclus* species from early to middle Miocene lacustrine sediments in Japan. (**A**) *A*. sp. 4. (**B, C**) *A*. *nipponicus*. (**D**) *A*. *bradburyii*. In **C**, a black arrow indicates a pseudonodulus.

Seven samples from three Lower and Middle Miocene lacustrine diatomaceous outcrops in Japan have yielded a total of four nonmarine *Actinocyclus* species, namely *Actinocyclus* sp. 1 (Figs 3A, 4A and 4I) from the Yamatoda Diatomaceous Mudstone Member (Nanao Formation) (Yamatoda-5 and 9 in Fig 2), *Actinocyclus* sp. 2 (Figs 3B, 3C, 4B, 4J and 4K) from the Chojabaru Formation (Chojabaru-19, 22 and 27 in Fig 2), *Actinocyclus* sp. 3 (Figs 3D, 4C, 4L, 4M and 4N) from the Chojabaru Formation (Chojabaru-5 in Fig 2) and *Actinocyclus* sp. 4 (Figs 3E, 4D and 5A) from the Ouchi Formation (Ouchi-1). Two *Actinocyclus* species, *A*. *nipponicus* and *A*. *bradburyii*, described from the RC12-394 core as new species [37], are shown in Figs 3F, 4E, 4H, 5B, 5C and 5D. In all samples, two genera, *Actinocyclus* and *Aulacoseira* Thwaites, are the dominant planktonic centric diatoms (Table 1), whereas Stephanodiscaceae genera are absent. Among pennate diatoms, a species with a linear valve detected in the sample Chojabaru-27 (relative abundance: 16.2%) resembles the extant *Fragilaria crotonensis* Kitton and hence may have been planktonic, whereas all the other samples yielded rare benthic species.

Detailed morphological descriptions of the four species of *Actinocyclus* will be presented in a future publication, but a concise description of the key characteristics of all four *Actinocyclus* species and *A*. *nipponicus* and *A*. *bradburyii* (Figs 4 and 5) is as follows: valve walls are characterized by fascicles composed of areola rows (Fig 4A–4H). The fascicles are sectioned by non-areolate hyaline stripes around the valve face/mantle area junction (Figs 4A, 4C, 4G, 4H, 4I, 5A and 5D). Individual areolae are externally obscured by a reticulate velum (i.e., cribrum) (Figs 4J, 5A, 5B and 5D). All species have a single pseudonodulus (Figs 4M and 5C) and many labiate processes (rimoportulae) (Fig 5C), which internally have a well-developed stalked labium and are located at the valve face/mantle area junction (Figs 4M and 5C). Valve walls of *A*. sp. 1, *A*. sp. 3 and *A*. sp. 4 are bullulate (bubbly) (Fig 4L), while that of *A*. sp. 2 is not (Fig 4K). These morphological characteristics, including the bullulate and non-bullate valve wall, are identical to those of fossil nonmarine *Actinocyclus* species reported from Lower to Middle Miocene lacustrine deposits in the western United States [13].

## Discussion

### Cenozoic continental planktonic diatoms

**Fig 6:**
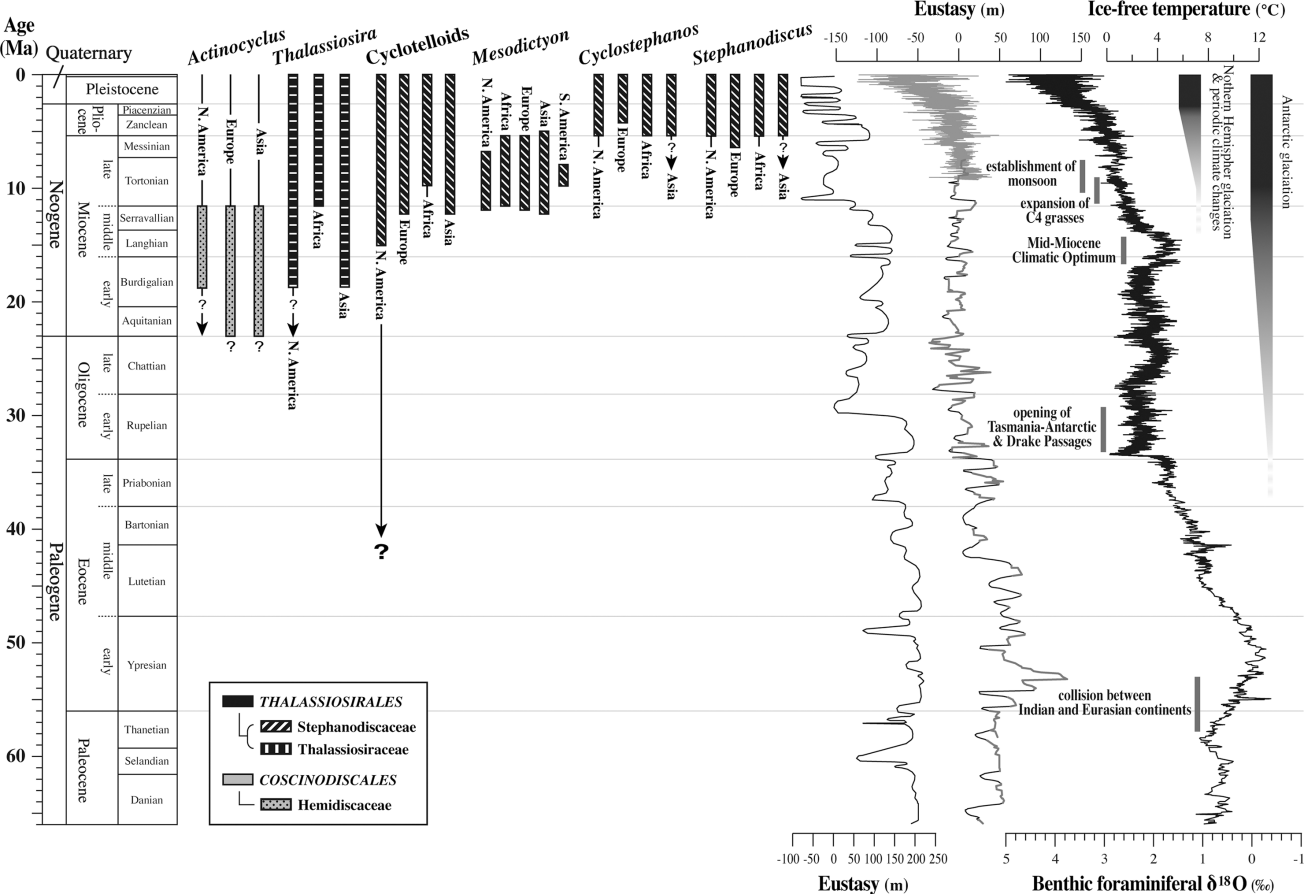
Comparison among geologic ranges of continental planktonic diatoms, eustatic sea-level changes and global climate changes. The geologic ranges of continental planktonic diatoms are modified from [4]. Cyclotelloids includes Stephanodiscaceae genera excepting *Cyclostephanos*, *Stephanodiscus* and *Mesodictyon*. Two eustatic curves (righ [48]; left [49]) have been calibrated to a recent geological time scale by [48]. Global climate changes are shown by a benthic foraminiferal δ^18^O record [50]. The chronostratigraphic chart follows [51].

A Cenozoic range chart for selected nonmarine planktonic diatom genera based upon biochronological data from several continents (Fig 6) reveals that the Miocene was an important time in the evolutionary history of continental planktonic diatoms. In eastern Asia, available data on early to middle Miocene nonmarine diatoms have been very limited, but it is now clear from this and a few recent studies based on SEM observations that at least eight nonmarine *Actinocyclus* species flourished there: *A*. *nipponicus* Hayashi, Saito-Kato & Tanimura, *A*. *bradburyii* Hayashi, Saito-Kato & Tanimura [37], *A*. *haradaae* (Pantoscek) Saito-Kato [46], *A*. *hiramakiensis* Tanaka [47], and the four species reported in this study. Recently, two studies based on LM observations also reported six additional possible species of nonmarine *Actinocyclus* from the Yamato Rise and the Ulleung Plateau in the Japan Sea: *A*. *cedrus* Bradbury & Krebs, *A*. *claviolus* Bradbury & Krebs, *A*. *nebulosus* Bradbury & Krebs, *A*. *gorbunovii* (Sheshukova) Moiseeva & Sheshukova, *A*. *krasskei* Bradbury & Krebs, *A*. *lobatus* (Rubina) Rubina & Khursevich [25, 26]. The dominance of *Actinocyclus* spp. in lakes during the early and middle Miocene in eastern Asia is therefore similar to the pattern observed in North America, Europe and northern Asia [4], indicating its global synchroneity (Fig 6). That is, obligate nonmarine *Actinocyclus* species first appeared in temperate lakes during the early Miocene, diversified during the middle Miocene, and disappeared by the end of the Miocene.

*Thalassiosira* Cleve was another important genus characterizing temperate nonmarine planktonic diatom assemblages during the early–middle Miocene [4]. Although the first marine species of *Thalassiosira* appeared during the middle Eocene [52], the first lacustrine species of *Thalassiosira* have been found in Lower Miocene rocks in North America and northern Asia [4] (Fig 6). Lacustrine species of *Thalassiosira* exist today, but they are usually rare, except in some east African rift lakes [9].

**Fig 7:**
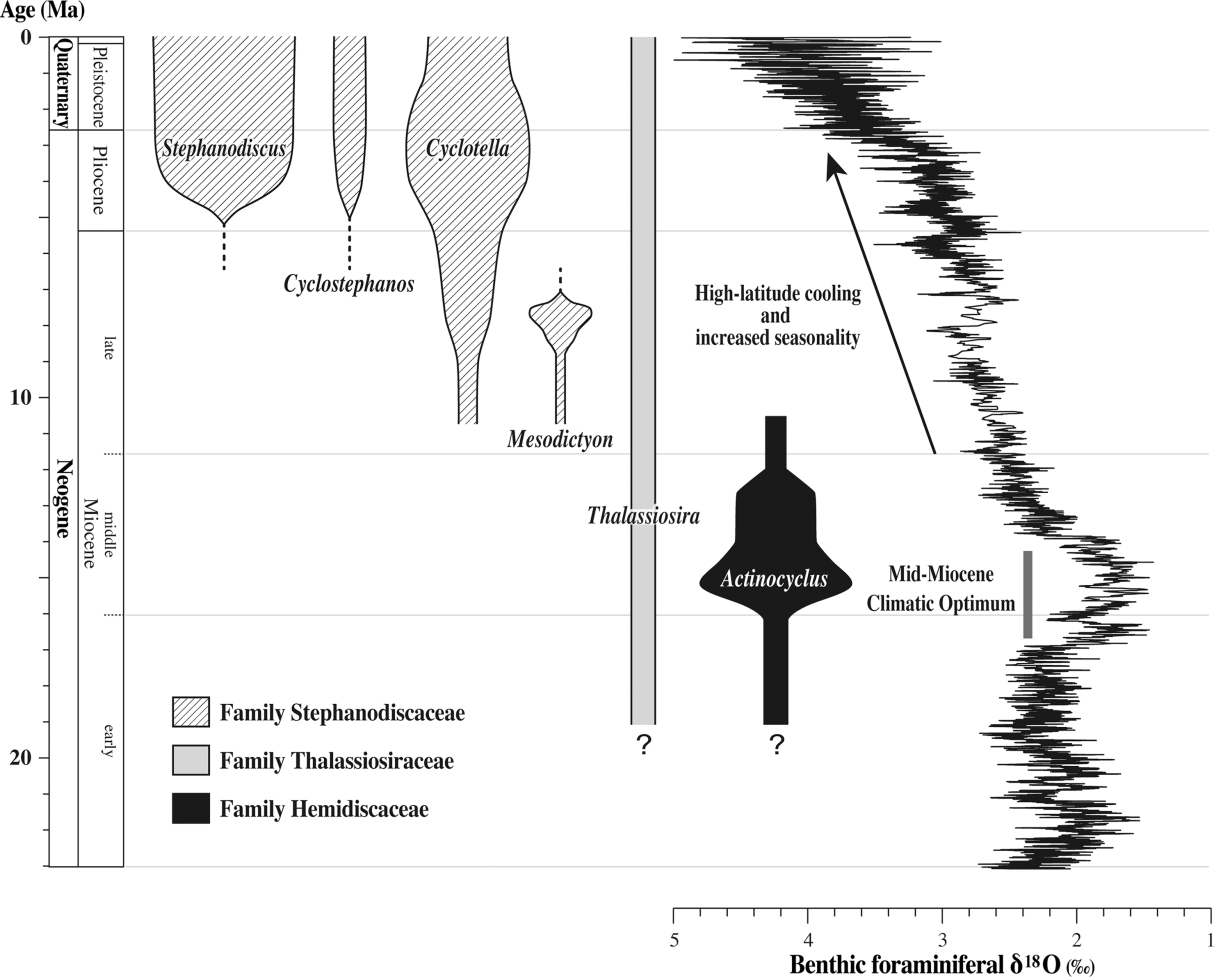
Biochronology with general relative diversities of six lacustrine diatom genera in mid-latitude North America and late Cenozoic global climate changes. The biochronology is modified from [5], which was established by radiometric dating of lacustrine diatomites. Diversity patterns are qualitative and not comparable between genera. The global climate record represented by benthic foraminiferal δ^18^O is after [50]. Time scale is from [51].

The late Miocene was a time when genera belonging to the family Stephanodiscaceae diversified, replacing species of nonmarine *Actinocyclus* at temperate latitudes [1, 4, 5] (Figs 6 and 7). *Mesodictyon* appears to be an extinct genus of Stephanodiscaceae restricted to the late Miocene. The occurrence of *Mesodictyon japonicum* Yanagisawa & Tanaka has been reported from the Lower Miocene lacustrine dredged samples from the Yamato Rise and the Ulleung Plateau based only on light microscopy [25, 26]. We suspect, however, that they are contaminants, re-deposited, or possibly misidentified because they are very rare [25, 26] and difficult to identify without SEM observation [25].

Another important event at temperate latitudes during the late Miocene was the burst in diversification of cyclotelloid species. The oldest cyclotelloids were once thought to have appeared in lower middle Miocene lacustrine rocks in northwest Nevada, USA [53] and in coeval or slightly older marine rock in California, USA [54]. Recently, however, middle Eocene cyclotelloids (*Cyclotella*, *Discostella* and *Puncticulata*) were reported in northern Canada [55], although an analysis, using Bayesian relaxed molecular clock methods, has cast doubt on the age of these occurrences [56]. Nevertheless, it appears that cyclotelloids did not become common and major constituents of temperate nonmarine planktonic diatom assemblages until the late Miocene.

At the beginning of the Pliocene, two extant obligate freshwater genera, *Cyclostephanos* and *Stephanodiscus*, began to diversify in temperate lakes [1, 4]. Since then, genera of Stephanodiscaceae (*Cyclostephanos*, *Stephanodiscus* and cyclotelloids) have been distinctive constituents of lake phytoplankton. In contrast, obligate nonmarine species of *Actinocyclus* have disappeared from lakes leaving the euryhaline *Actinocyclus normanii* f. *subsalsus* (Juhlin-Dannfelt) Hustedt as the only extant species adapted to lentic environments [57]. This turnover from nonmarine *Actinocyclus* assemblages to Stephanodiscaceae assemblages in temperate lakes near the middle/late Miocene boundary was an important event in the evolution of nonmarine diatoms that established the composition of today’s lacustrine diatom floras. In the next sections, we explore the global environmental changes that occurred during the Miocene which may have influenced diatom evolution and the morphological traits possessed by members of the Stephanodiscaceae that may have conferred a competitive advantage over *Actinocyclus*.

### Eustasy influencing continental planktonic diatom evolution

**Fig 8:**
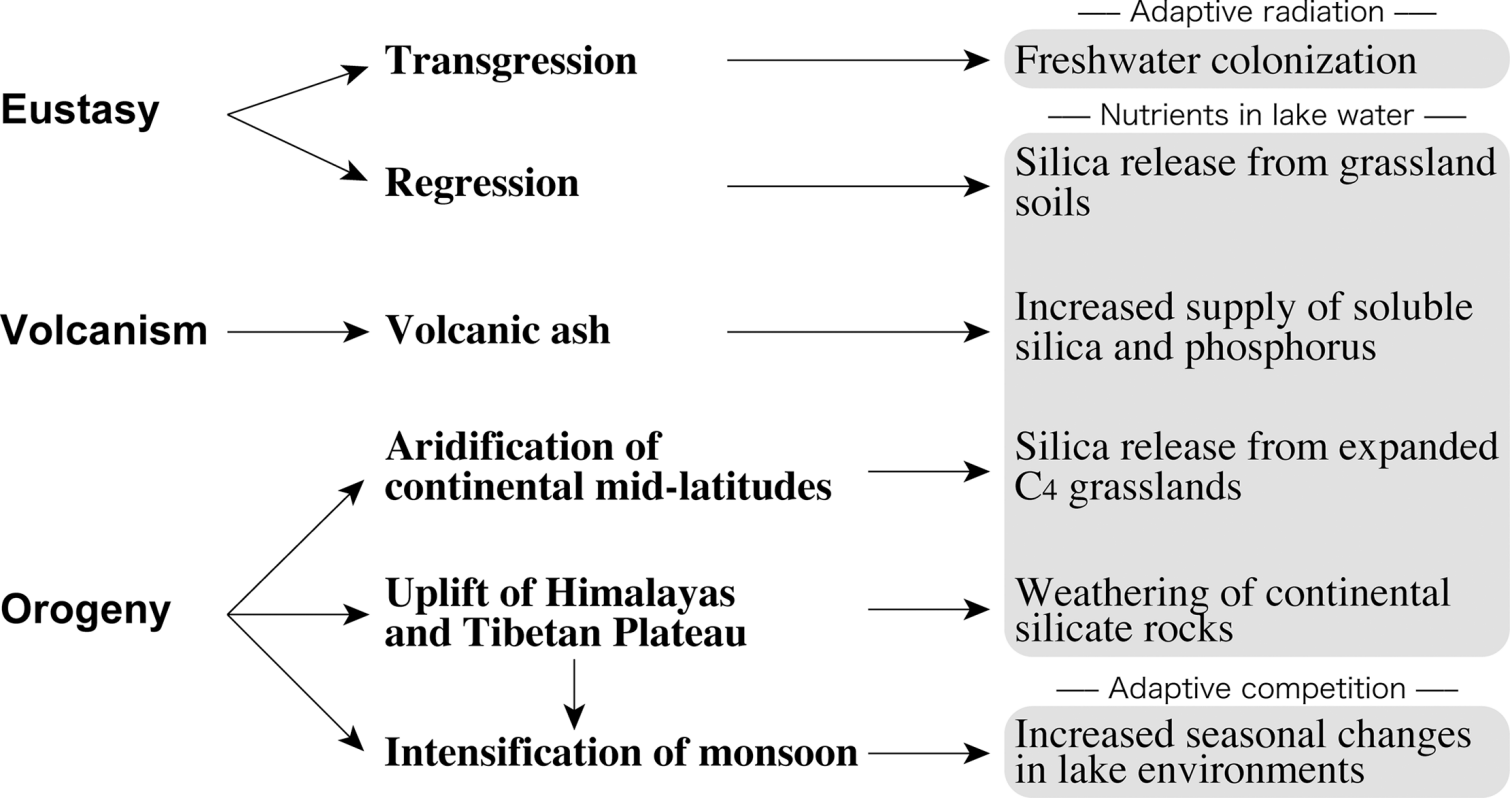
Potential effects of eustasy, volcanism, and orogeny on continental planktonic diatoms during the Neogene.

Despite limited data from the Southern Hemisphere, it appears that the pattern of evolution and turnover of temperate continental planktonic diatoms during the Neogene was global in scale. This near simultaneity of events must be attributed to large-scale climatic and environmental changes, and those of the Miocene are of particular interest. The maximum global warmth during the Neogene (Mid-Miocene Climatic Optimum) was attained at 15–17 Ma (Fig 7) and was followed by gradual cooling and increased seasonality through the late Neogene [27, 50]. The late Neogene cooling has been attributed to the development of the East Antarctic Ice Sheet and to enhanced meridional temperature gradients that followed the opening of the Tasmania–Antarctic Passage and the Drake Passage [27]. In addition, the Miocene is characterized by notable changes in eustasy, increased volcanism, and significant orogenies, and their consequent effects must have had a significant impact on continental biotas (Fig 8). The pattern of evolution and timing of turnovers of continental diatom assemblages during the Miocene appears to coincide with some of these events.

Although it has long been known that freshwater organisms are transported by wind, insects, and birds [58, 59], eustatic transgressions may also have facilitated the introduction of marine and brackish water species into lentic environments [4]. Rising sea level would have flooded river valleys, expanded estuarine environments, and caused water tables to rise, thereby promoting the transport of marine and brackish water organisms inland. *Actinocyclus* is documented in marine rocks as old as Eocene [60], but it was not until the latest Oligocene–earliest Miocene that the genus experienced a burst of speciation in marine waters [61, 62]. A highstand of sea level at that time [48, 49] (Fig 6) may have facilitated the introduction of euryhaline species into temperate lakes where obligate freshwater species evolved and diversified during the early and middle Miocene. Tectonism and volcanism in the Great Basin of the United States [63] and elsewhere in the world had created numerous lake basins at that time.

Genera of the family Stephanodiscaceae were believed to be monophyletic and to have evolved from nonmarine *Thalassiosira* during the latest middle Miocene (e.g., [3, 6]). This single freshwater colonization was thought to be rare and irreversible, comparable to Julius Caesar’s “crossing the Rubicon” by [64]. This hypothesis, however, is inconsistent with recent findings. First, the latest middle Miocene origin of the family Stephanodiscaceae conflicts with the possible discovery of middle Eocene taxa from the Giraffe Kimberlite Pipe in the Northwest Territories, Canada, whose morphological characteristics indicate that they apparently belong to Stephanodiscaceae genera *Cyclotella*, *Discostella* and *Puncticulata* [55]. Second, much earlier origins (Paleocene and Eocene, respectively) for the *Cyclotella* and cyclostephanoid lineages than previously believed have been suggested by a recent molecular clock study [56]. Finally, molecular phylogeny has demonstrated that genera of Stephanodiscaceae are really polyphyletic, originating from multiple freshwater colonization events [65–67]. According to [67], salinity imposes a barrier to the spatial distribution of some diatoms but is less formidable than previously believed. If so, it is possible that marine cold-water species of *Thalassiosira* and allied genera were introduced into high latitude lakes during Paleogene highstands of sea level [48, 49] where Stephodiscaceae genera evolved and diversified. Although the origins of nonmarine *Thalassiosira* and Stephanodiscaceae genera remain unresolved, it is evident from the accumulated fossil records that their great diversification and widespread dispersal in temperate latitudes began near the beginning of the late Miocene, consistent with the findings of the molecular clock study [56] that a large share of the extant species diversity of the genera of Stephanodiscaceae traces back to the middle Miocene.

### Increased lake silica

Increased availability of nutrients has often been invoked to explain the evolution, diversification, and increased productivity of both marine and nonmarine diatoms (e.g., [68–70]), and this may also have contributed to the turnover of temperate nonmarine planktonic diatoms near the middle/late Miocene boundary. The major nutrients necessary for diatom production are phosphate, nitrate and silica (e.g., [71]). Among these, silica may have been especially important because its availability has varied greatly (Fig 8). Volcanism has played a critical role in the formation of diatomaceous deposits because volcanic ash supplies soluble silica into lakes and thereby stimulates diatom productivity (e.g., [72, 73]). Additionally, volcanic ash may supply phosphorus for diatoms [20]. It has long been recognized that the global distribution of marine and nonmarine diatomites is commonly associated with the spatio-temporal distribution of volcanic rocks [70, 74–76], but it is also known that diatoms can flourish in lakes without volcanism (e.g., [73]). For example, planktonic diatoms that lived in the Paleo-Kathmandu Lake, which existed on the Kathmandu Basin on the southern slope of the central Himalaya, maintained high productivity through the middle to late Pleistocene without the association of volcanism [77], and this resulted in the accumulation of hundreds of meters of diatomaceous sediment [78]. The common association of lacustrine diatomites with volcanic rocks may simply reflect excellent preserving capacity because of increased resistance to erosion and the slowing of dissolution of biogenic opaline silica [73]. Even so, the role of volcanic ash as an important silica source for lake diatoms is undoubted.

Other factors can also contribute to increased silica in lakes (Fig 8). For example, orogeny has the potential to control the flux of silica into aquatic environments through the weathering of silicate rocks [68, 69, 79]. The greatest orogenic event during the Cenozoic was the uplift of the Himalayas and Tibetan Plateau [27]. They were gradually uplifted after the collision of India and Eurasia near the Paleocene/Eocene boundary [80], thus accelerating the weathering of silicate rocks in that region [81]. The late Miocene also coincided with enhanced uplift of the Sierra Nevada Mountains and adjacent Great Basin [82, 83] in North America and the Andes Mountains [84, 85] in South America.

Silicate dissolution was further facilitated by the worldwide expansion of C_4_ grasses [68, 69]. Opal phytoliths from C_4_ grasses are more soluble than abiotic silicate minerals [86], and they were readily transported into lakes through rivers, streams, and ground water, then dissolved, and the silica made available to diatoms [68, 69]. The expansion of C_4_ grasses was important because they provided a more widespread, sustained, and hence stable supply of silica for lake diatoms than episodic volcanism [70].

After the Mid-Miocene Climatic Optimum (see Fig 6), global climate experienced an increase in meridional temperature gradients, climatic zones, seasonality, and aridification of mid-latitude continental regions [87]. These changes promoted the evolution and expansion of grasslands [88]. In fact, the extensive expansion of C_4_ grasses during the late Miocene [89–91] coincided with the evolution and diversification of Stephanodiscaceae genera. Furthermore, silica dissolution may have accelerated during the numerous eustatic regressions that occurred after the late Miocene (Fig 6) because of increased soil erosion and phytolith dissolution during downcutting and expansion of river drainage systems [70].

Until now, we have summarized various factors that may have affected continental diatoms: eustatic changes of sea level, volcanism, the weathering of silicate rocks by the uplift of the Himalayas, the Tibetan Plateau and other mountainous regions, and the expansion of C_4_ grasses. Eustatic transgressions during the Cenozoic may have provided the opportunity for marine and brackish water species to colonize lacustrine habitats, followed by the enhanced availability of silica during the late Miocene that promoted diatom productivity. In fact, large amounts of both lacustrine and marine diatomaceous sediments accumulated during the late Miocene [70, 73, 92]. Nevertheless, those factors still may be insufficient to account for the turnover of nonmarine temperate planktonic diatom assemblages near the middle/late Miocene boundary. For example, the increased availability of silica during the late Miocene might have stimulated diatoms such as *Actinocyclus* species already adapted to lake environments. To account for the turnover of diatom assemblages at that time, another environmental factor that caused a change in the usage of the increased silica must have occurred.

### Adaptation to periodic environmental changes

Aside from environmental changes during the Neogene, the functional morphology of planktonic lacustrine diatoms may have played a role in their evolution. In fact, the genus *Actinocyclus* of the family Hemidiscaceae and genera of the family Stephanodiscaceae have distinctly different morphological characteristics. The most noticeable morphological difference is the strutted process (fultoportula), which is present in genera of Stephanodiscaceae, but absent in *Actinocyclus* [71]. Species in the family Stephanodiscaceae have strutted processes not only on the mantle but often on the valve face. β-chitin fibrils released from the valve face strutted processes link with adjacent cells in colony formation, and those from the mantle strutted processes create more surface area, both of which serve to facilitate buoyancy [93]. Thus, species of Stephanodiscaceae genera, both solitarily free living and forming colonial chains, increased their ability to float, drift, and remain in the photic zone longer than *Actinocyclus* species.

**Fig 9:**
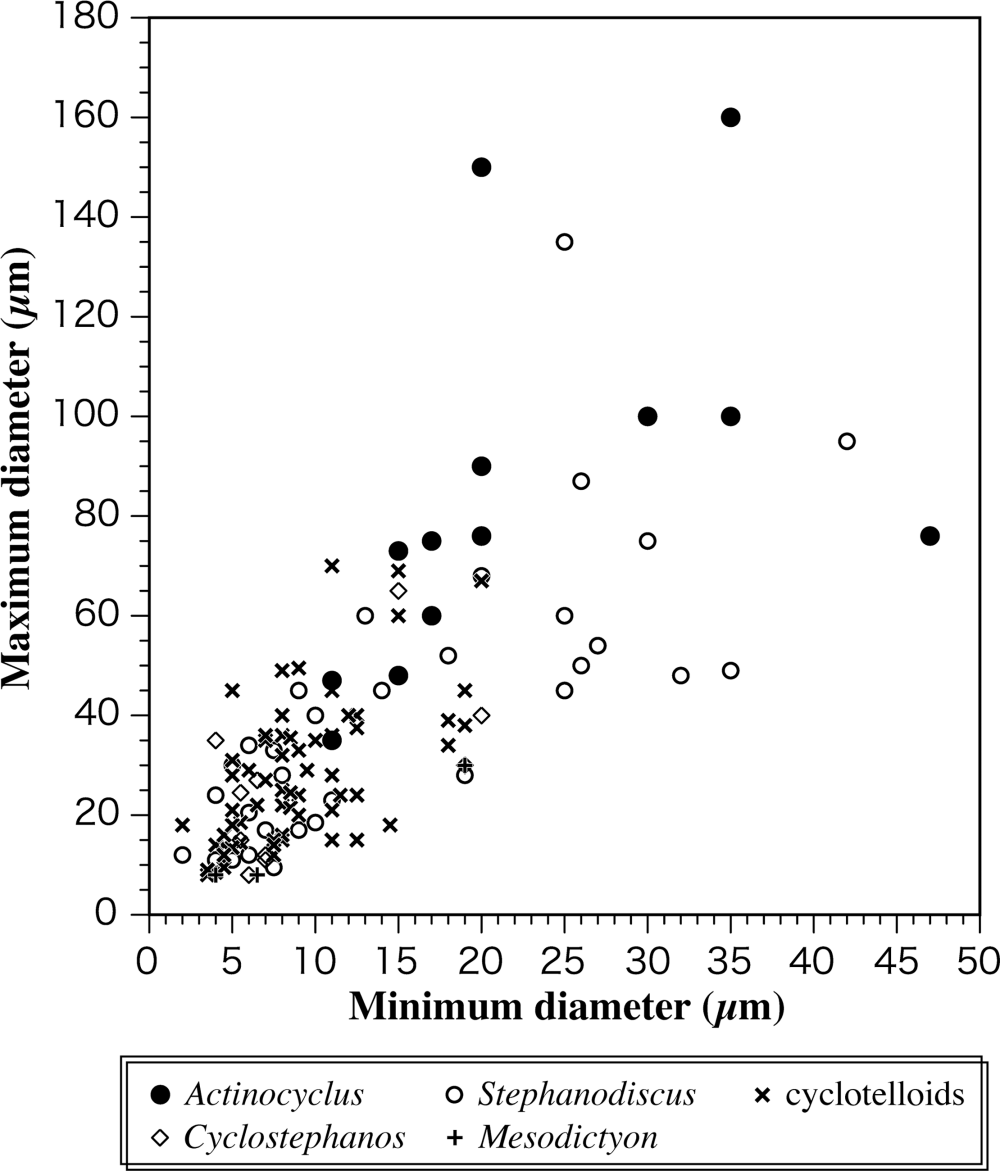
Comparison of valve diameter among nonmarine. ***Actinocyclus*, *Cyclostephanos*, *Stephanodiscus*, *Mesodictyon* and cyclotelloid species.** Data are collected from [13, 37, 43, 94, 97].

The other important morphological difference between species of Stephanodiscacae and *Actinocyclus* species is cell volume. In general, cell volumes of species in the family Stephanodiscaceae are much smaller than those of nonmarine *Actinocyclus* species (Fig 9). For example, the maximum valve diameter of most species of Stephanodiscaceae is less than 50 μm, except large *Stephanodiscus niagarae* Ehrenberg and a few related species (e.g., [43, 94]). In contrast, maximum valve diameter of most species of nonmarine *Actinocyclus* is greater than 50 μm [13]. In modern oceans, diatoms (e.g., *Chaetoceros* Ehrenberg, *Skeletonema* Greville) associated with high productivity in coastal regions and in the seas around Antarctica tend to be small in size, lightly silicified, and chain forming [95, 96], similar to Stephanodiscaceae genera.

Cell (or body) size of an organism has been used to predict metabolic rate based on the size-scaling exponent (3/4 law; Kleiber's rule [98, 99]). According to the classic 3/4 law, smaller cell size of species in Stephanodiscacae genera may reflect a higher metabolic rate than *Actinocyclus* species. Some recent studies, however, suggest the metabolic rate of marine diatoms shows a nearly isometric scaling relationship with cell volume and thus deviates from the 3/4 law [100–103], because of, for example, the package effect of chlorophyll-specific absorption [104–106]. Small cell size may confer another advantage based upon the r/K selection theory [107]. In general, the r-strategists, characterized by small body size, show a high intrinsic growth rate and early reproduction [107, 108]. Because genera of Stephanodiscaceae originated from marine *Thalassiosira*, a typical r-strategist [109, 110], they may have inherited that trait. Although much remains unknown about the ecological significance of phytoplankton cell volume, the significant difference in valve size between Stephanodiscacae genera and *Actinocyclus* [Fig 9] may reflect differences in physiology and ecology.

Species of the family Stephanodiscaceae, having strutted processes and being generally much smaller than *Actinocyclus* species, may have had a selective advantage in floating and drifting capabilities as well as higher rates of metabolism, intrinsic growth, and reproductivity. These may have facilitated the rapid utilization of nutrient resources, especially if those nutrients were seasonally limited by wind induced upwelling. We believe that this advantage may have spurred their evolution from thalassiosiroid ancestors and caused the turnover of nonmarine planktonic diatoms at temperate latitudes near the middle/late Miocene boundary. If true, then the evolution of periodic, particularly seasonal, climatic and environmental changes that have increased since the late Miocene may provide an explanation for the turnover and diversification of Stephanodiscaceae. In addition, because seasonality is greater at high latitudes, Stephanodiscaceae genera may have first evolved there during the Paleogene and then spread to lower latitudes during the latest middle and late Miocene as climates deteriorated, ultimately displacing *Actinocyclus* spp.

We suspect that the development of monsoons may also have been an important piece of this puzzle because they play a major role in seasonal changes in wind and nutrient loading in modern lakes. In modern times, monsoons dominate climatic and environmental conditions in Asia, Australia, Africa and lower latitude regions of North and South America [111–113] and can exercise a strong influence regionally by other means [e.g., the El Niño-Southern Oscillation (ENSO)] (e.g., [114, 115]). Monsoon is defined as seasonally reversing winds: wet sea wind during summer months; dry land wind during winter months [111]. Wind is a primary force to move lake water at all depths by means of surface drift, surface waves, Langmuir spirals, surface and internal seiches, the seasonal turnover of lake water, etc. [116, 117]. Therefore, the seasonally reversing monsoon winds caused turbulent mixing and turnover of lake water, contributing to the upwelling of nutrients into the photic zone as well as the re-suspension of meroplanktonic (temporarily planktonic) diatoms in the water column. In addition, the amount of nutrients available for lacustrine diatoms should have increased due to heavy monsoon rainfall because it accelerates nutrient loading from the land into lakes. In summary, planktonic lacustrine diatoms (particularly Stephanodiscaceae species) that were well adapted to seasonal fluctuations in lake environments may have possessed selective advantages during the late Miocene as monsoon conditions evolved and intensified.

Geological evidence and simulation results from global climate models have revealed that Asian monsoon systems developed as a result of the uplift of the Himalayas and Tibetan Plateau [118–125]. The Himalayas and Tibetan Plateau attained sufficient elevation at about 10 Ma to establish monsoon systems, and by ~7.5 Ma had attained their current intensity [123, 124, 126–131]. The late Miocene development of monsoons is known to have initiated various kinds of faunal and floral changes (e.g., [89, 132–136]), and their inception at ca. 10 Ma approximates the turnover of nonmarine planktonic diatoms from nonmarine *Actinocyclus* assemblages to Stephanodiscaceae assemblages. The intensification of Asian monsoons at ca. 7.5 Ma coincides with the diversification of Stephanodiscaceae genera (Fig 6). We speculate that the floral turnover of nonmarine planktonic diatoms near the middle/late Miocene boundary and diversification of Stephanodiscaceae genera at temperate latitudes were influenced by the development of monsoons and the concomitant increase in silica loading into lakes.

The late Cenozoic evolution of Stephanodiscaceae genera may have been further influenced by other climatic and environmental events. For example, the transition from the early middle Miocene to late Miocene coincided with the intensification of significant high latitude cooling [27, 50], increased meridional temperature gradients [87], uplift of the Sierra Nevada and Andes mountains [82–85], and the evolution of modern terrestrial ecosystems [87]. In particular, increased seasonality due to enhanced meridional temperature gradients should also have necessitated new survival strategies for lacustrine diatoms. In northern latitudes, spring coincided with both increased light availability and onset of winds that disturbed thermally stratified lake waters, allowing nutrients that had accumulated in bottom waters to rise to surface waters where they could stimulate diatom productivity. Additionally, seasonality and various time-scale periodicity of Asian monsoon may have coevolved with glacial-interglacial cycles on orbital time scales (e.g., [27, 137]), stadial-interstadial cycles on millennial to centennial scales (e.g., [138–141]) and the Indian Ocean Dipole and ENSO on decadal to annual scales (e.g., [142–144]) during the latest Cenozoic. Although they are different in both their temporal and spatial distributions, all of them have been closely associated with monsoon activity (e.g., [77, 144, 145]). Therefore, the development of periodic changes in global and regional climate systems and associated monsoon conditions may have facilitated the species diversification of Stephanodiscaceae genera by compelling them to adapt to rapid changes in lake environments during the late Cenozoic.

## Conclusions

Lacustrine planktonic diatom floras during the early to middle Miocene in eastern Asia were characterized by nonmarine *Actinocyclus* species, consistent with those at temperate latitudes in North America, Europe, Africa, and northern Asia. The evolution and turnover of temperate nonmarine planktonic diatoms at the middle/late Miocene boundary coincided with climatic and environmental changes associated with eustasy, volcanism, and orogeny. It is possible that the eustatic transgression during the latest Oligocene–earliest Miocene enabled marine and brackish water *Actinocyclus* species to colonize and subsequently diversify in temperate lacustrine habitats during the early and middle Miocene. Similarly, thalassiosiroid ancestors of Stephanodiscaceae genera may have been introduced into high latitude lakes during Cenozoic highstands of sea level, accounting for the polyphyletic origins of the family. The middle/late Miocene turnover in temperate lakes from *Actinocyclus* to Stephanodiscaceae assemblages coincided with increased silica loading into lakes by active volcanism, increased weathering of silicate rocks due to orogeny (e.g., Himalayas and Tibetan Plateau) and expansion of C_4_ grasslands. In addition, increased seasonality from the establishment of monsoon systems and high latitude cooling during the late Miocene advantaged Stephanodiscaceae genera because of their superiority in floating and drifting capabilities, and perhaps metabolism, intrinsic growth rate, and reproductivity. As seasonal conditions expanded, those genera were transported to lower latitudes where they diversified, eventually displacing *Actinocyclus*. Future research, such as the investigation of high latitude fossil lacustrine diatoms and Paleogene lacustrine diatomaceous rocks everywhere, is needed to test these hypotheses. Another effective approach may be in a study of *Aulacoseira*, the other major constituent of planktonic lacustrine diatom assemblages during the middle to late Cenozoic.
